# MLH1 deficiency leads to deregulated mitochondrial metabolism

**DOI:** 10.1038/s41419-019-2018-y

**Published:** 2019-10-22

**Authors:** Sukaina Rashid, Marta O. Freitas, Danilo Cucchi, Gemma Bridge, Zhi Yao, Laura Gay, Marc Williams, Jun Wang, Nirosha Suraweera, Andrew Silver, Stuart A. C. McDonald, Claude Chelala, Gyorgy Szabadkai, Sarah A. Martin

**Affiliations:** 1 Centre for Molecular Oncology, Barts Cancer Institute, Queen Mary University of London, Charterhouse Square, London, EC1M 6BQ UK; 2 Department of Cell and Developmental Biology, Consortium for Mitochondrial Research, University College London, London, WC1E 6BT UK; 3 Centre for Tumour Biology, Barts Cancer Institute, Queen Mary University of London, Charterhouse Square, London, EC1M 6BQ UK; 4 Blizard Institute, Barts and the London School of Medicine and Dentistry, Queen Mary University of London, 4 Newark Street, London, E1 2AT UK; 5 Department of Biomedical Sciences, University of Padua, Padua, 35131 Italy; 6 The Francis Crick Institute, London, NW1 1AT UK

**Keywords:** Cancer metabolism, Cell biology

## Abstract

The DNA mismatch repair (MMR) pathway is responsible for the repair of base–base mismatches and insertion/deletion loops that arise during DNA replication. MMR deficiency is currently estimated to be present in 15–17% of colorectal cancer cases and 30% of endometrial cancers. MLH1 is one of the key proteins involved in the MMR pathway. Inhibition of a number of mitochondrial genes, including POLG and PINK1 can induce synthetic lethality in MLH1-deficient cells. Here we demonstrate for the first time that loss of MLH1 is associated with a deregulated mitochondrial metabolism, with reduced basal oxygen consumption rate and reduced spare respiratory capacity. Furthermore, MLH1-deficient cells display a significant reduction in activity of the respiratory chain Complex I. As a functional consequence of this perturbed mitochondrial metabolism, MLH1-deficient cells have a reduced anti-oxidant response and show increased sensitivity to reactive oxidative species (ROS)-inducing drugs. Taken together, our results provide evidence for an intrinsic mitochondrial dysfunction in MLH1-deficient cells and a requirement for MLH1 in the regulation of mitochondrial function.

## Introduction

When the genes that mediate the DNA mismatch repair (MMR) pathway, such as *MLH1*, *MSH2* and *MSH6*, are mutated or epigenetically silenced, the predisposition to cancer is vastly increased^1^. In particular, germline mutations in the MMR genes *MLH1* and *MSH2* predispose to Lynch syndrome^2^. MMR deficiency is present in numerous tumour types including colorectal and endometrial cancers^1,3,4^. Specifically, MLH1 expression is lost in 8–21% of colorectal cancers^5–7^ and 24–37% of endometrial cancers^4,8,9^.

Mitochondria are essential organelles in all eukaryotic cells that mediate cellular energy (adenosine triphosphate (ATP)) production via oxidative phosphorylation. During this process, electrons are transferred through a series of oxidative phosphorylation complexes known as the electron transport chain (ETC) in which a proton gradient is produced across the inner mitochondrial membrane to form an electrochemical membrane potential^10^. This membrane potential is then used by the F_0_F_1_ ATP synthase to generate ATP. Importantly, mitochondria are also major sites of reactive oxidative species (ROS) production. Therefore, unsurprisingly mitochondrial dysfunction is detrimental to the cell. For example, ROS produced via accidental escape of electrons from the oxidative phosphorylation complexes I and III can induce oxidative damage to lipids, proteins and DNA^11^. Indeed, mitochondrial dysfunction is implicated in the pathology of numerous diseases including cancer. Although the main role of the MMR pathway is the repair of DNA replication errors, there is evidence that it has several non-canonical roles, including participating in homologous recombination, mitotic and meiotic recombination, and in the repair of oxidative DNA damage^12–14^. More recently, a role has been suggested for MLH1 in the mitochondria. We and others have previously shown that MLH1 can localise to the mitochondria and inhibition of a number of mitochondrial genes, including POLG and PINK1, can induce synthetic lethality in MLH1-deficient cells^14–17^. This synthetic lethal interaction was associated with an increase in oxidative DNA lesions (8-oxoG) in the mitochondrial DNA (mtDNA).

mtDNA is particularly prone to oxidative DNA damage for a variety of reasons, including its close proximity to the ETC where the majority of ROS is generated and the fact that it is not protected by histones^18^. It is estimated that the levels of oxidative damage in the mitochondria are two to three times higher than in nuclear DNA^19,20^. It has been established that mitochondria utilise base excision repair as their primary mechanism for repairing mitochondrial oxidative DNA damage^21^. Nevertheless, there is increasing evidence that some form of MMR machinery is present in the mitochondria and that MMR proteins are potentially also involved in the repair of oxidative DNA damage to mtDNA^22–24^.

Herein, we provide evidence that MLH1 is required for the maintenance of mitochondria function. We elucidate how targeting mitochondrial function may be a novel therapeutic approach for the treatment of MLH1-deficient disease.

## Results

### MLH1 loss is associated with decreased mitochondrial bioenergetics

**Fig. 1:**
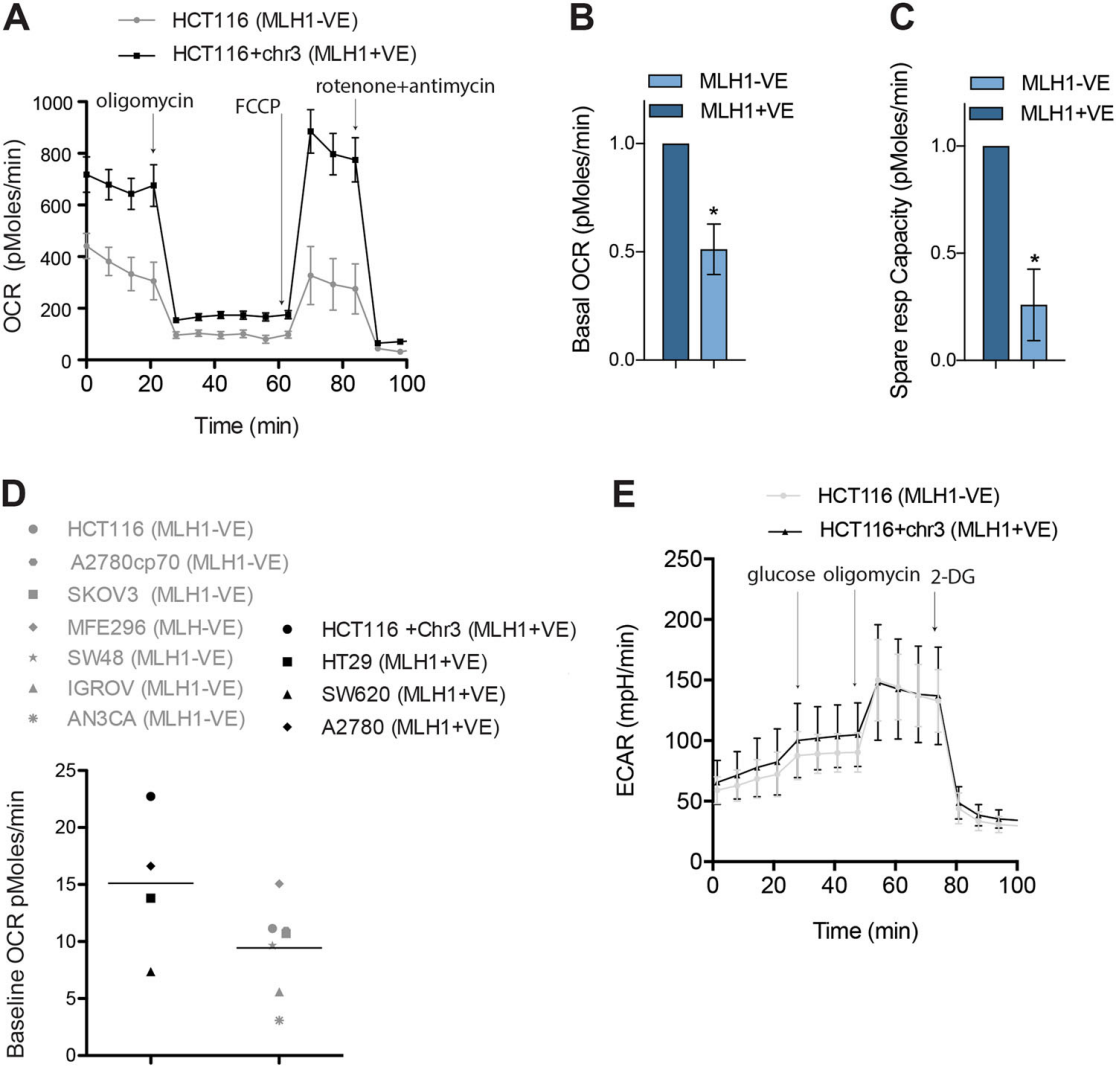
MLH1 loss is associated with decreased oxygen consumption rate and reduced spare respiratory capacity. The Seahorse Bioscience XF24 extracellular flux analyser was used to measure OCR (pMoles/min), indicative of OXPHOS in MLH1-deficient HCT116 cell line and the MLH1-proficient HCT116+ chr3 cell line. **a** After establishing a baseline, oligomycin (1 μM), FCCP (0.25 μM), rotenone (1 μM) and antimycin (1 μm) were sequentially added, as indicated by arrows. **b** The basal OCR was calculated using the difference between the mean of time points in baseline and in oligomycin treatment (baseline minus oligomycin OCR). **c** The spare respiratory capacity was calculated as (OCR following FCCP—baseline OCR). **d** The basal OCR was lower in a panel of MLH1-deficient cell lines (HCT116, SKOV3, IGROV, AN3CA, MFE-296, SW48 and A2780cp70) compared to the MLH1-proficient cell lines (HCT116+ chr3, HT29, SW620 and A2780). **e** After establishing a baseline, glucose (10 mM), oligomycin (1 μM) and 2-DG (50 mM) were sequentially added, as indicated by arrows. **b–d** Experiments were carried out in triplicate and error bars represent standard error of the mean (SEM). *p < 0.05

Our previous studies have suggested that inhibition of a number of mitochondrial genes is synthetically lethal with MLH1 loss^14,17^. Therefore, we hypothesised that mitochondrial function may be altered in MLH1-deficient cells. To investigate this further, we determined initially whether mitochondrial bioenergetics are deregulated in MLH1-deficient cells. To this end, we analysed oxygen consumption rates (OCR) and the extracellular acidification rate (ECAR) in the MLH1-deficient colorectal cancer cell line, HCT116 and the isogenically matched MLH1-proficient, HCT116+ chr3 cells, using the Seahorse XtraFlux (XF) analyser. The XF analyser measures the rate of oxygen consumption in a given sample providing a measure of oxidative phosphorylation. The basal OCR represents a measure of basal oxidative phosphorylation and upon addition of the uncoupling agent, carbonyl cyanide-p-trifluoromethoxyphenylhydrazone (FCCP), a measure of the cells maximal respiratory capacity is estimated. When the basal OCR is subtracted from the maximal respiration, a measure of spare respiratory capacity (SRC) is obtained. The SRC reflects the cells ability to respond to stress and increased energy demands. Interestingly, we observed a decrease in the basal OCR (Fig. 1a, b; *p* < 0.05) and a decrease in the SRC (Fig. 1c; *p* < 0.05) in the MLH1-deficient cells compared to the MLH1-proficient cells. To determine whether these differences were specific to the HCT116 isogenic colorectal cancer cell lines, we measured the basal OCR of a panel of MLH1-proficient and MLH1-deficient cell lines from a range of different tumour types and a range of different genetic backgrounds. We observed a decrease in the basal OCR upon loss of MLH1 regardless of tumour cell type (Fig. 1d). The impact of MLH1 loss on glycolysis was determined by analysing the ECAR of the surrounding media, by measuring the excretion of lactic acid per unit time after its conversion from pyruvate. No significant difference was observed in the ECAR in the MLH1-deficient colorectal cancer cell line, HCT116 in comparison to the isogenically matched MLH1-proficient, HCT116+ chr3 cells (Fig. 1e). Taken together, our results show for the first time that MLH1 loss is associated with decreased oxidative phosphorylation and with a reduced capacity to respond to increased energy demands.

### Decreased Complex I activity in MLH1-deficient cells

**Fig. 2:**
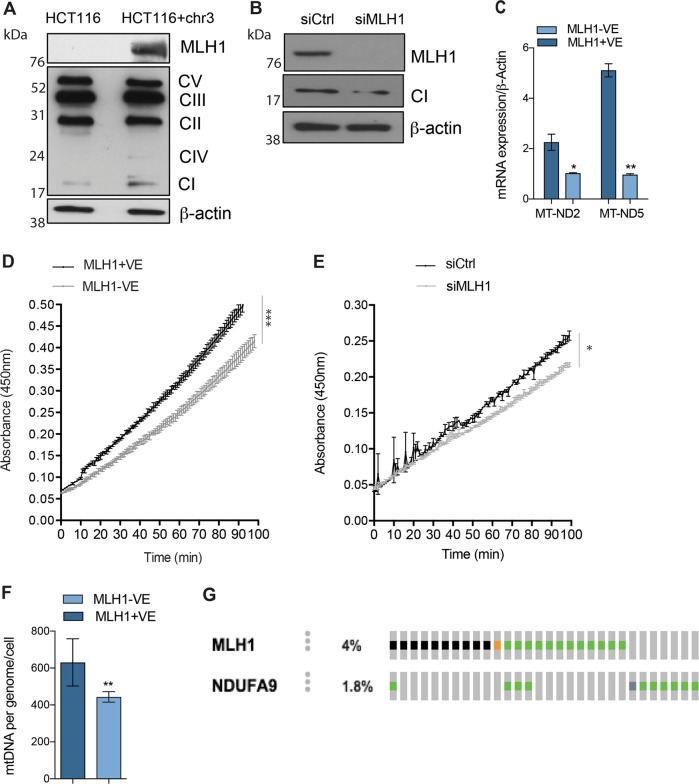
Reduced Complex I activity in MLH1-deficient cells. **a** Western blot analysis of MLH1-deficient HCT116 and MLH1-proficient HCT116+ chr3 cells. Protein was extracted and expression was analysed using anti-Total OXPHOS, anti-MLH1 and β-actin antibodies. β-actin is used as a loading control. **b** Western blot analysis of MLH1-proficient KLE cells transfected with either non-targeting control siRNA (siCtrl) or siRNA targeting MLH1 (siMLH1). Protein was extracted and expression was analysed using anti-Complex I (accessory subunit NDUFB8), anti-MLH1 and β-actin antibodies. β-actin is used as a loading control. **c** Quantitative RT-PCR analysis of RNA extracted from HCT116 and HCT116+ chr3 cells. mRNA expression was measured using ND2, ND5 and β-actin Taqman probes. β-actin was used as a control. Complex I activity was measured using an ELISA assay. Protein lysates were isolated from the **d** MLH1-deficient HCT116 and MLH1-proficient HCT116+ chr3 cell lines and **e** KLE cells transfected with either siControl or siRNA targeting MLH1. Equal amounts of protein were incubated to determine the activity of Complex I by measuring the oxidation of NADH to NAD+ and the simultaneous reduction of a dye leading to increased absorbance at 450 nm, over time. **f** Reduced mtDNA copy number in MLH1-deficient cells. Relative mtDNA copy number expressed as a ratio of total genomic DNA in MLH1-deficient HCT116 and MLH1-proficient HCT116+ chr3 cells. **g** Analysis of data from 619 colorectal adenocarcinoma patient samples. Mutations in MLH1 and NDUFA9, as represented using OncoPrint analysis from cBioPortal, where each bar is representative of a tumour that contains a mutation. Aligned bars represent the same tumour. Green lines represent missense mutations, orange lines represent in frame mutations, black lines represent known truncating mutations (putative driver mutations) and grey lines represent known truncating mutations (putative passenger mutations). The incidence of MLH1 mutation was 4%, whilst the incidence of NDUFA9 mutation was 1.8% in the 619 patient samples. *NDUF9A* was mutated in 17.39% of *MLH1* mutated cases, in comparison to 1.17% of *MLH1* wild-type cases. All experiments were carried out in triplicate and error bars represent the SEM. *p < 0.05, **p < 0.005. See also Fig. S1A–C

To investigate further the decreased oxidative phosphorylation observed in our panel of MLH1-deficient cells, we assessed the expression levels of the five oxidative phosphorylation complexes in our cells. We observed decreased expression of Complex I, in the MLH1-deficient HCT116 cell line in comparison to the MLH1-proficient cell line HCT116+ chr3 (Fig. 2a; Supplementary Fig. 1A; *p* < 0.0005). We further validated our observation by analysing Complex I expression in the MLH1-proficient endometrial cancer cell line KLE, transfected with either Control, non-targeting siRNA or siRNA targeting MLH1 (Fig. 2b; Supplementary Fig. 1B; *p* < 0.05). Complex I expression was also reduced upon MLH1 silencing. To determine whether this reduction was specific to the accessory subunit NDUFB8 measured or Complex I in general, we analysed gene expression of the mitochondrial-encoded Complex I subunits *MTND2 and MTND5* (Fig. 2c; *p < 0.05, **p < 0.005). Our data demonstrate that expression of *MTND2* and *MTND5* were also decreased in the HCT116 MLH1-deficient cells, in comparison to the MLH1-proficient cells. To further investigate Complex I in the absence of MLH1 expression, we next measured whether MLH1 expression is required for Complex I activity. Complex I activity was measured using an ELISA assay assessing the oxidation of NADH to NAD+. Interestingly, we observed a decrease in the activity of Complex I in the MLH1-deficient cell line HCT116, compared to the MLH1 proficient HCT116+ chr3 cells (Fig. 2d; *p* < 0.0005). To ensure this reduction was not specific to HCT116 cells, we analysed Complex I activity upon MLH1 silencing in the MLH1-proficient KLE cells (Fig. 2e; *p* < 0.05). We also observed a reduction in Complex I activity upon MLH1 silencing. In addition, we analysed Complex I activity in our diverse panel of MLH1-deficient and -proficient cells and observed a statistically significant decrease in the activity of Complex I upon MLH1 loss across all cell lines (Supplementary Fig. 1C). Our results suggested that oxidative phosphorylation was impaired in MLH1-deficient cells due to decreased Complex I activity. Previous studies have reported that colorectal cancer cells can have increased mutations within microsatellites in mitochondrial-encoded Complex I genes^23^. It is widely known that MLH1-deficient cells have increased nuclear microsatellite instability (MSI), therefore we hypothesised that perhaps the deregulated OCR and abrogated Complex I activity, we observe in our MLH1-deficient cells may be due to MSI in mitochondrial-encoded Complex I genes. To elucidate this further, we carried out next-generation sequencing (NGS) on the mitochondrial genome of the HCT116 and HCT116+ chr3 cells, using the Illumina MiSeq platform. Interestingly we did not observe any differences in mutations within the seven known mitochondrial-encoded Complex I genes as well as the non-coding D-loop regions, which are known to harbour mutations affecting Complex I (Supplementary Table 1). We only identified one coding mutation, which was in the MLH1-proficient cells in MT-CO1 gene at a very low frequency (~26%). However, it is of note that the NGS analysis we performed was limited with regards to MSI detection, and therefore our MSI analysis was inconclusive. We next looked at mitochondrial copy number in the MLH1-deficient and -proficient cells by determining the ratio of the mitochondrial *tRNA* gene, relative to the nuclear housekeeping gene, *β2M*. Interestingly, there was a striking decrease in the mitochondrial copy number upon MLH1 loss (Fig. 2f). Our data suggest that in the absence of MLH1, replication of mtDNA is impaired.

### Mutations in the Complex I subunit NDUFA9 significantly co-occur with MLH1 mutations in colorectal adenocarcinoma patients

Our results indicate that Complex I activity is perturbed in our panel of cell lines from a range of tumour types. We next investigated if similar Complex I dysfunction was also observed in MLH1-deficient patient tumour samples. To this end, we interrogated the whole exome sequencing data from 619 cases of colorectal adenocarcinoma patients^25^ using the cBioPortal tool (www.cbioportal.org). We observed that the Complex I subunit *NDUFA9* was mutated in 17.39% of *MLH1* mutated cases in comparison to 1.17% of *MLH1* wild-type cases (Fig. 2g; Table 1). This finding indicates that there is a significant co-occurrence (*p* = 0.00004) of *MLH1* loss and *NDUFA9* mutations in colorectal adenocarcinoma patients, therefore supporting our in vitro data.

**Table 1:** Significant co-occurrence of MLH1 and NDUFA9 mutations identified upon whole exome sequencing of 619 colon adenocarcinoma patient samples (25)

Gene	Cytoband	Percentage of alteration (*MLH1* mut)	Percentage of alteration (*MLH1* wt)	Log ratio	*p* Value	*q* Value	Tendency
*NDUFA9*	12p13.32	4 (17.39%)	7 (1.17%)	3.89	4.05E-04	0.0251	Co-occurrence

### Reduced expression of mitochondrial biogenesis and anti-oxidant defence genes in MLH1-deficient cells

**Fig. 3:**
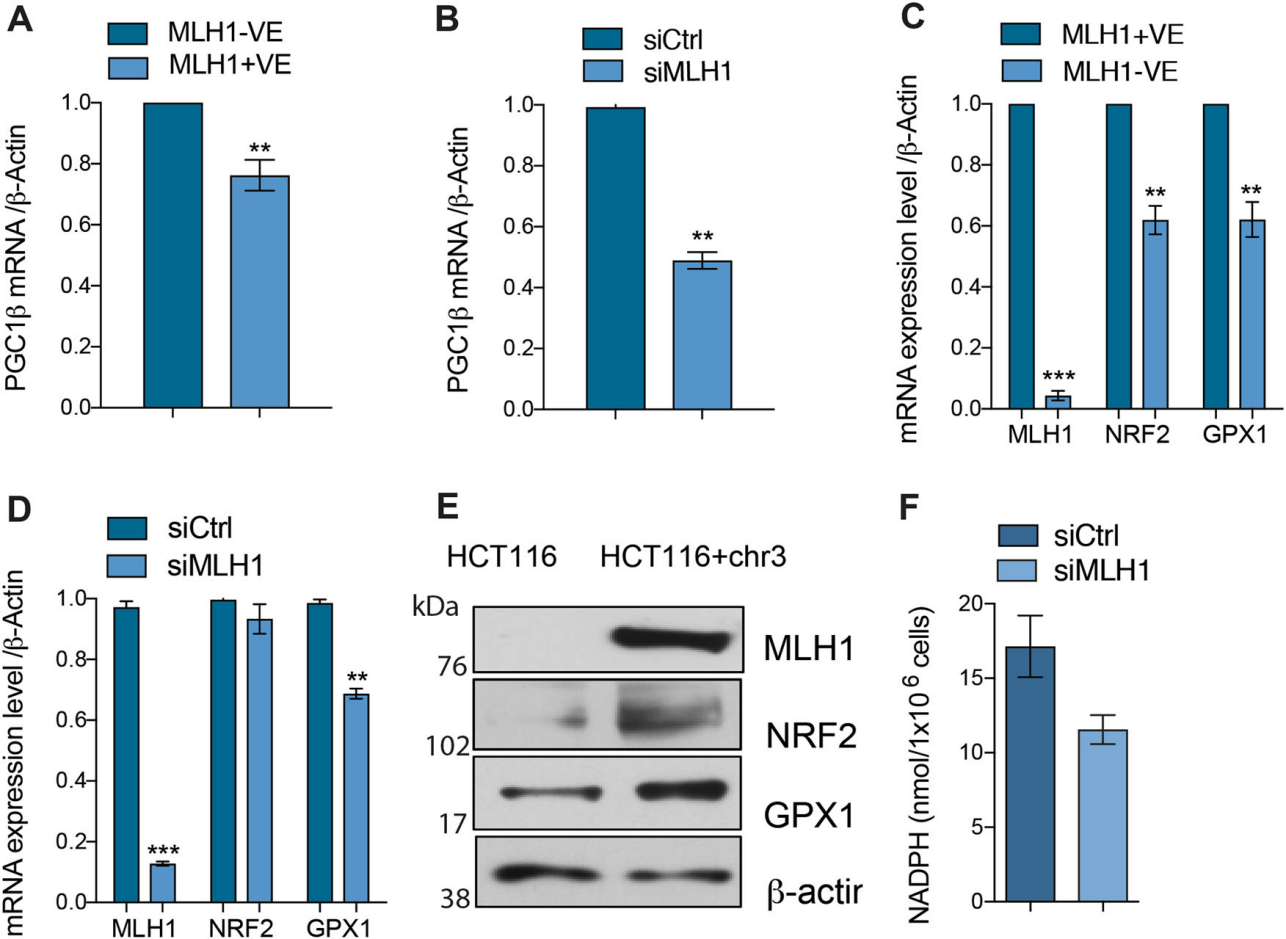
MLH1 loss is associated with reduced mitochondrial gene expression. Quantitative RT-PCR analysis of RNA extracted from **a** HCT116 and HCT116+ chr3 cells or **b** KLE cells transfected with either siCTRL siRNA targeting MLH1. mRNA expression was measured using PGC1β and β-actin Taqman probes. β-actin was used as a control. Quantitative RT-PCR analysis of RNA extracted from **c** HCT116 and HCT116+ chr3 cells or **d** KLE cells transfected with either siCTRL or siRNA targeting MLH1. mRNA expression was measured using MLH1, NRF2, GPX1 and β-actin Taqman probes. β-actin was used as a control. **e** Western blot analysis of MLH1-deficient HCT116 and MLH1-proficient HCT116+ chr3 cells. Protein was extracted and expression was analysed using antibodies as indicated. β-actin is used as a loading control. **f** GPX1 activity was measured using a commercially available GPX1 activity assay. Protein lysates were isolated from the KLE cells transfected with either siControl or siRNA targeting MLH1. Equal amounts of protein were incubated to determine the activity of GPX1 by measuring the optical density for NADPH at 340 nm before and after addition of cumene hydroperoxide (substrate for GPX1)

Our results thus far have described a mitochondrial phenotype in MLH1-deficient cell lines, with deficiencies in Complex I, decreased oxidative phosphorylation and reduced mtDNA copy number. Therefore, we next investigated whether mitochondrial biogenesis, in general was attenuated upon MLH1 loss. To this end, we examined the expression of the nuclear transcriptional co-activator PGC-1β in the MLH1-deficient and -proficient cell lines, since PGC-1β is known as the master regulator of mitochondrial biogenesis^26^. We observed a decrease in the expression of *PGC-1β* mRNA in the MLH1-deficient HCT116 cell line (Fig. 3a; *p* < 0.005) and upon MLH1-silencing in the MLH1-proficient KLE cells (Fig. 3b; *p* < 0.005). We have previously shown that increased oxidative DNA damage to mtDNA is synthetically lethal with MLH1 deficiency^14,17^. A recent study has shown that reduced levels of the antioxidant response protein, NRF2 can lead to decreased Complex I levels^27^. Given our observations that Complex I activity is reduced upon MLH1 loss and MLH1-deficient cells are sensitive to increased oxidative DNA damage, we hypothesised that loss of MLH1 was associated with a reduced antioxidant response. To elucidate this, we analysed whether expression of proteins involved in the antioxidant response were altered in the absence of MLH1 expression. NRF2 is the key transcription factor and master regulator of the antioxidant defence pathway. It detects signals activated due to cellular oxidative stress and activates downstream genes including glutathione peroxidase 1 (*GPX1*). To determine whether our MLH1-deficient cells have an aberrant antioxidant response, we measured the expression of the antioxidant response genes *NRF2* and *GPX1* in our MLH1-deficient and -proficient cells and upon MLH1 silencing in the MLH1-proficient KLE cells. Interestingly, we observed a decrease in expression at both the RNA (Fig. 3c, d; **p < 0.005, ***p < 0.0005) and protein (Fig. 3e) level. To further investigate this, we measured the activity of GPX1 in the MLH1-proficient KLE cells transfected with either siControl or siRNA targeting MLH1 (Fig. 3f). We observed a reduction in GPX1 activity upon MLH1 silencing.

Taken together, our data suggest that MLH1-deficient cells have a broad rearrangement of mitochondrial gene expression including decreased mitochondrial biogenesis and anti-oxidant response genes, leading to a dysfunctional mitochondrial phenotype.

### MLH1 loss results in increased sensitivity to the Complex I inhibitor, Rotenone

**Fig. 4:**
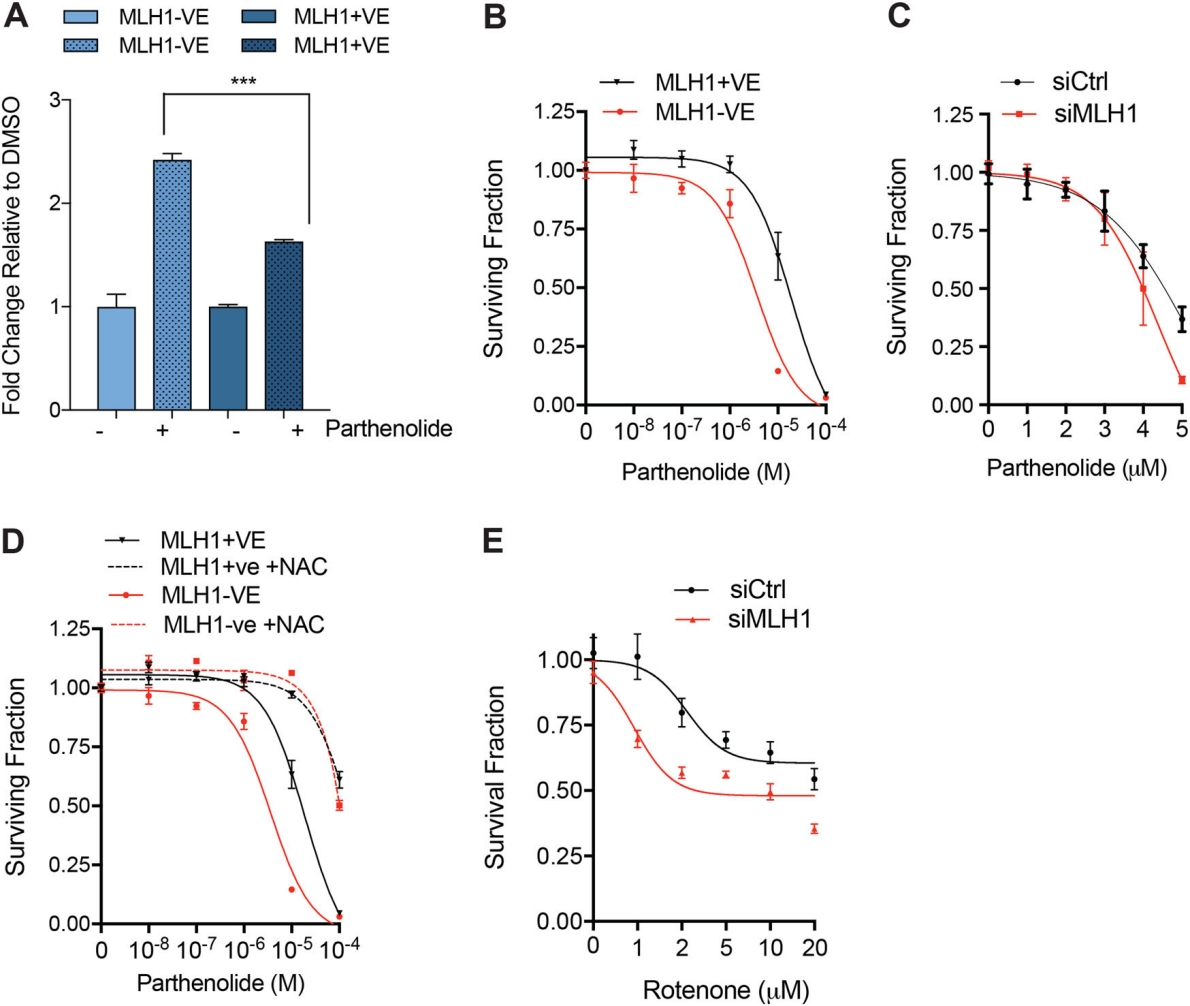
MLH1 loss is associated with increased ROS and sensitivity upon Parthenolide treatment. **a** Increased ROS levels in MLH1-deficient cells upon Parthenolide treatment. The HCT116 and HCT116+ chr3 cells were treated with either DMSO or Parthenolide (10 μM) for 30 min. Cells were washed and treated with 10 µM DHE, and fluorescence was measured on the MitoXpress fluorescent microscope as a measure of ROS production. **b** HCT116 and HCT116+ chr3 cells or **c** KLE cells transfected with either siControl or siRNA targeting MLH1, were treated with increasing concentrations of the Parthenolide. After 4 days treatment, cell viability was measured using an ATP-based luminescence assay. **d** The HCT116 and HCT116+ chr3 cells were treated with increasing concentrations of the Parthenolide, in the presence or absence of N-acetyl cysteine (NAC). After 4 days treatment, cell viability was measured using an ATP-based luminescence assay. **e** KLE cells transfected with either siControl or siRNA targeting MLH1, were treated with increasing concentrations of the Rotenone. After 4 days treatment, cell viability was measured using Alamar Blue. All experiments were carried out in triplicate and error bars represent the SEM. *p < 0.05, **p < 0.005, ***p < 0.0005. See also Fig. S1D

Our results suggest that expression of the anti-oxidant response genes, NRF2 and GPX1 are reduced in MLH1-deficient cells. Given that MLH1 loss gives rise to a reduced antioxidant response, we investigated whether this attenuated response resulted in sensitivity to increased oxidative stress. To this end, we first treated the HCT116 and HCT116+ chr3 cells with the ROS-inducing agent Parthenolide (Fig. 4a, b). It has been previously shown that Parthenolide activates NADPH oxidase and causes oxidative stress by inducing ROS^28^. We observed that the MLH1-deficient HCT116 cells were more sensitive to Parthenolide treatment, in comparison to the MLH1-proficient HCT116+ chr3 cells (Fig. 4b). In addition, we observed that silencing of MLH1 sensitised the MLH1-proficient KLE cells to Parthenolide treatment (Fig. 4c). To determine whether it was oxidative stress that was causing this selectivity, we treated our cell lines with increasing concentrations of Parthenolide, in addition to the ROS scavenging agent N-acetyl-cysteine (NAC; Fig. 4d). Addition of NAC rescued the selectivity observed with Parthenolide treatment in MLH1-deficient cells, therefore suggesting that it is oxidative stress that results in the reduced cell viability in HCT116 cells upon Parthenolide treatment. To further assess the generality of our observations, we examined Parthenolide sensitivity in a panel of MLH1 deficient and proficient tumour cell lines (Supplementary Fig. 1B). Although *MLH1* mutations are not the only genetic variable within this diverse tumour cell panel, we observed a clear distinction in Parthenolide sensitivity between tumour lines with wild‐type MLH1 expression and those with MLH1 deficiency. We also observed increased sensitivity of MLH1-deficient cells to the ROS-inducing, Complex I inhibitor, Rotenone (Fig. 4e). Our results therefore suggest that the decreased ability of MLH1-deficient cells to respond to oxidative stress has uncovered a vulnerability that may be exploited therapeutically by ROS-inducing agents.

**Fig. 5:**
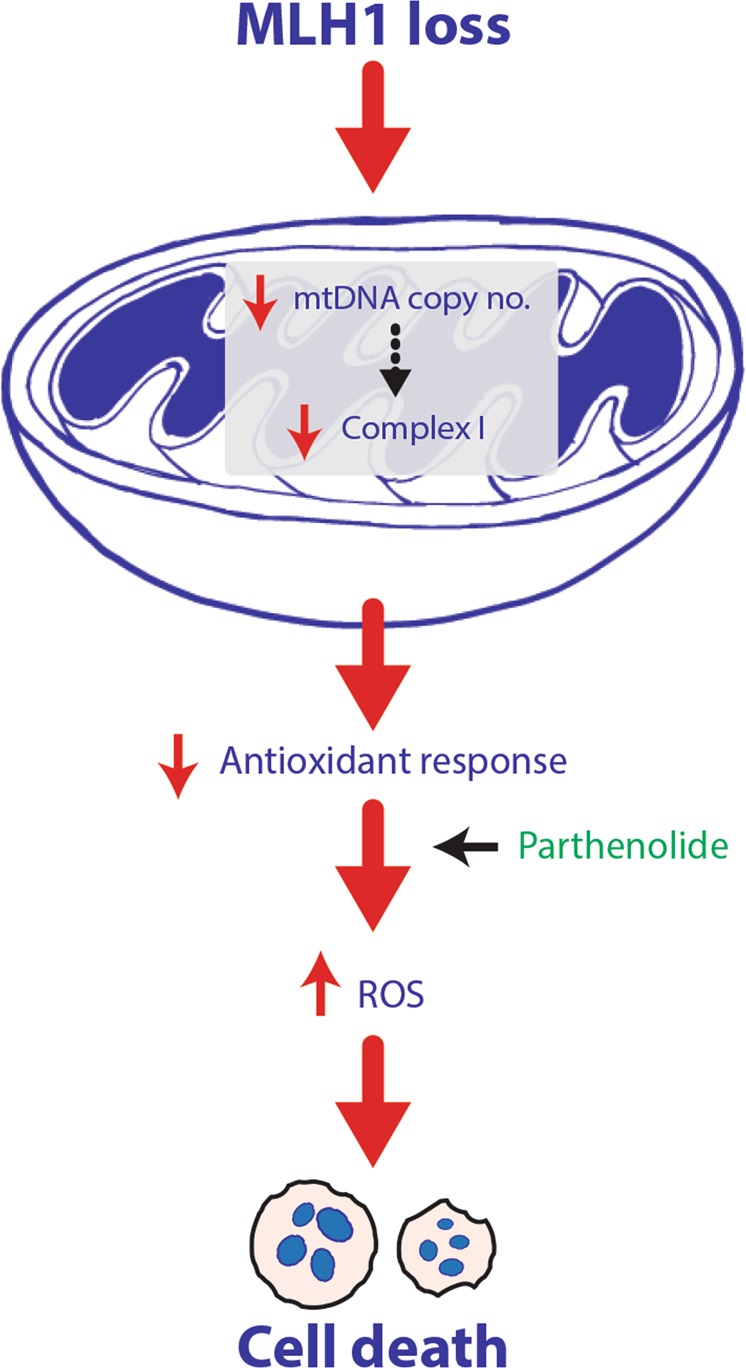
Schematic illustration of mitochondrial dysfunction and subsequent increased sensitivity to Parthenolide in MLH1-deficient cells. MLH1-deficient cells display a reduced mtDNA copy number, dysfunctional Complex I activity and a reduced anti-oxidant stress response leading to the sensitivity of MLH1-deficient cells to increased oxidative stress

Taken together, our data indicates that impaired Complex I activity and a subsequent perturbed anti-oxidant stress response is responsible for the sensitivity of MLH1-deficient cells to increased oxidative stress (illustrated in Fig. 5).

## Discussion

Here, we show for the first time a novel role for MLH1 in maintaining functional mitochondria. Given the dual function of MLH1 in repair of DNA replication errors and mitochondria biogenesis, it is inherently difficult to dissect a separate impact of these two roles upon tumourigenesis. The mutator phenotype is clearly the driving force behind carcinogenesis in many MMR-deficient tumours but does impaired mitochondrial metabolism also contribute, either via increased oxidative mtDNA damage or independently? A number of studies have examined the specific role of oxidative damage repair by the MMR pathway in relation to tumourigenesis. Colussi et al.^29^ demonstrated that a decrease in 8-oxoG levels translated into a decrease in the MMR-mediated mutator effect, by expressing MTH1 in MSH2-deficient mouse embryonic fibroblasts. In addition, treating MLH1-deficient cells in the presence of the antioxidant ascorbate, with and without H_2_O_2_ treatment, reduced mutation rates and reduced MSI by 30%^30^. However, studies have also suggested that MLH1 deficient, HCT116 cells are less sensitive to H_2_O_2_ than their MMR proficient counterparts (HCT116+ chr3)^30,31^. We have previously shown that MSH2-deficient cells are more sensitive to treatment with H_2_O_2_^32^. Furthermore, treatment with the ROS-inducing agent, potassium bromate in an *Msh2*^−/−^ mice, increased the formation of epithelial tumours in the small intestine^33^. The evidence thus far suggests that the MMR system may suppress carcinogenesis in the context of oxidative damage by directly repairing ROS-induced DNA lesions or acting as a sensor of oxidative damage, thereby activating apoptosis.

Loss of the MMR pathway is currently estimated to occur in approximately 30% of endometrial tumours. Loss of MMR has been associated with high tumour grade and metastasis in endometrial cancer^34^. Here, we show for the first time that loss of MLH1 results in reduced OCR, reduced Complex I activity, and increased reactive oxygen species. Previously, it has been shown that mtDNA mutations that resulted in reduced Complex I activity and increased ROS enhanced the metastatic potential of tumour cells^35^. Given that loss of MMR is a key feature of metastasis in endometrial cancer, it would be interesting to understand whether the mitochondrial phenotype driven by MLH1 loss gives rise to the increased metastasis in MMR-deficient endometrial cancer.

The DNA repair protein Ataxia-telangiectasia (ATM) is recruited to sites of DNA damage resulting in DNA repair, apoptosis or cell cycle arrest^36,37^. There is emerging evidence that the phenotype observed in ataxia-telangiectasia is unlikely to be solely related to the nuclear DNA repair functions of ATM and that ATM has a role in the mitochondria. Studies have shown that A–T lymphoblastoid cells (ATM-deficient) have dysfunctional mitochondria compared to wild-type cells as evidenced by abnormal mitochondrial structure, reduced mitochondrial membrane potential and decreased mitochondrial respiration^38^. In addition, ATM-deficient thymocytes have swollen mitochondria with abnormal cristae in addition to increased mitochondrial ROS and decreased Complex I activity^39^. ATM signalling has been shown to be involved in the regulation of ribonucleotide reductase (RR), the rate-limiting enzyme essential for the synthesis of deoxyribonucleoside triphosphates and mitochondrial homoeostasis, and therefore ATM is required for the control of mtDNA copy number in response to oxidative DNA damage^40^. ATM-deficient cells have lower levels of NAD+ due to the persistent unrepaired DNA damage thus these cells have a reduced antioxidant capacity and increased ROS levels^41^. It is well established that when the MMR pathway is recruited to sites of DNA damage, MLH1 can associate with ATM in recruiting other components of the DNA damage response pathway^42^. Therefore, the role of MLH1 in maintaining mitochondrial function may be in part associated with ATM. It may be possible that the interaction of ATM with MLH1 is required to maintain mitochondrial homoeostasis.

Taken together, we have elucidated a vulnerability in MLH1-deficient tumour cells such that due to dysfunctional mitochondria, these cells have a reduced antioxidant response. Collectively, our data suggest that this decrease in the antioxidant response and deregulated mitochondrial metabolism drives sensitivity to the Complex I inhibitor Rotenone, in MLH1-deficient cells and can be exploited clinically.

## Experimental procedures

### Cell culture

The human colon cancer cell line HCT116 and HCT116+ chr3 were a kind gift from Dr. Alan Clark (NIEHS). The human ovarian cancer cell lines A2780, A2780cp70, SKOV3 and IGROV were a kind gift from Dr. Michelle Lockley (QMUL). The human endometrial cancer cell lines, KLE, MFE-296 and AN3CA, and the human colon cancer cell lines, HT29 and SW48 were purchased from ATCC. All cell lines were grown in DMEM (Sigma-Aldrich), 10% foetal calf serum (FBS; Invitrogen) and 100 U/ml penicillin and 100 µg/ml streptomycin at 37 °C/5% CO_2_ apart from SKOV and IGROV which were routinely grown in RPMI-1640 media (Sigma) supplemented with 10% FBS and 100 U/ml penicillin and 100 µg/ml streptomycin at 37 °C/5% CO_2_. All cell lines were authenticated on the basis of short tandem repeat-profile, viability, morphologic inspection, and were routinely mycoplasma tested. Parthenolide and Rotenone were purchased from Sigma-Aldrich. NAC was purchased from Santa Cruz.

### Cell viability assays

Cells were seeded in 96-well plates (1–2 × 10^3^ cells/well) 24 h before treatment with increasing concentrations of Rotenone or Parthenolide. After 5 days, cell viability was assessed using either CellTiter Glo (Promega) or Alamar Blue (Invitrogen).

### Western blot

Cells were lysed in 20 mM Tris (pH 8), 200 mM NaCl, 1 mM EDTA, 0.5% (v/v) NP40, 10% glycerol, supplemented with protease inhibitors (Roche). Equivalent amounts of protein were electrophoresed on 4–12% Novex precast gels (Invitrogen) and transferred to nitrocellulose membrane. After blocking for 1 h in 1×TBS/5% non-fat dried milk, membranes were incubated overnight at 4 °C in primary antibody, including anti-MLH1 (#4256, Cell Signalling), anti-Total OXPHOS Human Antibody Cocktail (Ab110411, Abcam), anti-NRF2 (Ab137550, Abcam), anti-GPX1 (#3286, Cell Signalling) and anti-β-actin-HRP (#4970, Cell Signalling). Membranes were incubated with anti-IgG-horseradish peroxidase and visualised by chemiluminescent detection (Supersignal West Pico Chemiluminescent Substrate, Pierce). Immunoblotting for β-Actin was performed as a loading control.

### Real-time quantitative PCR (qRT-PCR)

RNA was extracted from cells using an RNeasy kit (Qiagen) and quantified using a Nanodrop spectrophotometer (Thermo). The Omniscript cDNA synthesis kit (Qiagen) was used to reverse transcribe 500 ng of RNA and 1 µl cDNA was used in each quantitative polymerase chain reaction (PCR) reaction. Multiplex PCR was performed on the ABI Prism 7500 Real-Time PCR Instrument (Applied Biosystems) and the δδCt method was used for data analysis. Taqman probes for each indicated target were purchased from Applied Biosystems (Life Technologies).

### siRNA transfections

For siRNA transfections, KLE cells were transfected with an MLH1 siRNA (Qiagen) using Lipofectamine RNAiMax (Invitrogen) according to the manufacturer’s instructions. As a control for each experiment, cells were transfected with a non-targeting control siRNA and concurrently analysed.

### Complex I activity

Complex I activity was measured using the Complex I activity ELISA assay (ab109721, Abcam), according to manufacturer’s instructions. Samples were added to a microplate pre-coated with capture antibodies specific to Complex I. After the target was immobilised, Complex I activity was determined following the oxidation of NADH to NAD+ and the simultaneous reduction of the provided dye, which leads to increased absorbance at OD 450 nm.

### GPX1 activity

GPX1 activity was measured using the GPX1 activity assay (ab102530, Abcam), according to manufacturer’s instructions. Samples were washed with PBS, resuspended in Assay Buffer, homogenised and centrifuged for 15 min at 10,000*g* at 4 °C. Assay plates were set up with supernatants and standards. NADPH, glutathione reductase and glutathione solutions were added to samples for 15 min to deplete all glutathione disulphide. Optical density for NADPH at 340 nm was measured before and after addition of cumene hydroperoxide (substrate for GPX1).

### Seahorse extracellular flux analyser

OCR, ECAR and SRC were measured using the Seahorse extracellular flux analyser (Seahorse Bioscience). A calibration plate (Seahorse Bioscience) containing a sensor cartridge with ports to allow addition of drugs was placed on top of a calibration plate, which was hydrated by adding 1 ml of calibrant solution (Seahorse Bioscience) to each well of the calibrant plate. Cells were seeded in XF 24-well or 96-well cell culture microplates (Seahorse Bioscience) and incubated for 24 h, growth media was removed from each well and replaced with 500 µl of assay medium (pH 7.4) pre-warmed to 37 °C. The cells were incubated at 37 °C for a least 60 min in a non-CO_2_ incubator to allow media temperature and pH to reach equilibrium before the first rate measurement. Prior to each rate measurement, the XF24 or XF96 Analyser gently mixed the assay media in each well to allow the oxygen partial pressure to reach equilibrium. Following mixing, OCR was measured to establish a baseline rate. The assay medium was then gently mixed again between each rate measurement to restore normal oxygen tension and pH in the microenvironment surrounding the cells. Uncoupled, maximal and non-mitochondrial respiration was determined after the addition of oligomycin (1 μM), FCCP (0.25 μM), rotenone (1 μM) and antimycin (1 μm). ECAR was determined after the addition of glucose (10 mM), oligomycin (1 μM) and 2-DG (50 mM). All chemicals were purchased from Sigma.

### ROS analysis

ROS levels were measured by high content fluorescence imaging, using the ImageXpress Micro XL (Molecular Devices) instrument. Cells were plated in a 96-well black/clear bottom plate (BD Falcon) and after 24 h treated with either DMSO or Parthenolide (1 µM) and incubated for 30 min at 5% CO_2_. Cells were then incubated with dihydroethidium (DHE), (Invitrogen, 10 µM). Cytosolic DHE exhibits blue fluorescence and upon reaction with superoxide anions, becomes oxidised to 2-hydroxyethidium, intercalates with DNA, and exhibits a red fluorescence (excitation/emission 530/380 (nm))^43^. The rate of the increase in flurescence, which reflects probe oxidation was measured in the same number of sites and cells per well using the ImageXpress instrument. Each experiment was carried out in triplicate and a ratio between the fluorescence values at 530 and 380 nm were calculated for each sample and data plotted is the average increased ratio rate.

### mtDNA copy number quantification

The measurement of mtDNA copy number, relative to nuclear DNA copy number was determined by amplifying mitochondrial tRNA-Leu(UUR) and nuclear-encoded β2m (beta-2-microglobulin) genes. Total DNA was extracted using the QIAmp DNA mini kit (Qiagen, Hilden, Germany), according to the manufacturer’s instructions. RT PCR reactions were performed on a LightCycler® 480 RT PCR Instrument (Roche Diagnostic). Totally, 2 μl of total DNA (3 ng/μl) and primer pairs in a total volume of 25 μl, were added to Light Cycler® 480SYBR Green I Master Mix (Roche Diagnostics). Human β2m forward (5′-TGCTGTCTCCATGTTTGATGTATC T-3′) and β2m reverse (5′-TCTCTGCTCCCCACCTCTAAGT-3′) or tRNA-Leu(UUR) forward (5′-CACCCAAGAACAGGGTTTGT-3′) and tRNA-Leu(UUR) reverse (5′-TGGCCATGGGTATGTTGTTA-3′) primers were used. The following protocol was used: 50 °C for 2 min, 95 °C for 10 min, 40 cycles of 95 °C for 15 s and 62 °C for 60 s. A dissociation curve was also calculated for each sample to ensure presence of a single-PCR product. To determine the mtDNA content, relative to nuclear DNA the following equations were used: ΔCT = (nucDNA CT − mtDNA CT); relative mtDNA content = 2 × 2^ΔCT^.

### mtDNA sequencing

Total DNA extraction was carried out for each cell line in triplicate, using the QIAamp DNA extraction kit (Qiagen) according to the manufacturer’s instruction. mtDNA was amplified in two overlapping fragments using the MTL-1 (9065 bp) and MTL-2 primers (11170 bp; Illumina) with high-fidelity Takara LA Taq (Clontech). Reactions were performed in duplicate. DNA products were checked for the correct size and quantified for subsequent normalisation to 0.2 ng/μL using a 2200 TapeStation instrument (Agilent). Dual indexed libraries were generated from 1 ng of PCR products using Nextera XT library preparation technology (Illumina) and sequenced on the Illumina MiSeq v2 platform, according to the manufacturer’s recommendations. The MiSeq re-sequencing protocol for small genome sequencing was followed according to the manufacturer’s recommendations. On-board software (i.e., Real-TimeAnalysis and MiSeq Reporter) converted raw data to Binary Alignment/Map and Variant Call Format v4.1 files using Genome Analysis Toolkit. The sequenced region of interest was aligned to the revised Cambridge Reference Sequence. Each nucleotide position was interrogated and variations from the reference were annotated by base difference. The median sequencing depth was 9261×.

### Statistical analysis

Unless stated otherwise, data represent standard error of the mean of at least three independent experiments. The two-tailed paired Student’s *t* test was used to determine statistical significance with *p* < 0.05 regarded as significant.

## Supplementary information

Supplementary Table 1

Supplementary Figure Legends

Supplementary Figure 1
