# Supplementary material for: MLH1 deficiency leads to deregulated mitochondrial metabolism
Source: Cell Death Dis. 2019 Oct 22;10(11):795. doi: 10.1038/s41419-019-2018-y (PMC6805956; doi:10.1038/s41419-019-2018-y)
Supplement: Supplementary file 1 — Supplementary Table 1 [file 41419_2019_2018_MOESM1_ESM.docx]

**Supplementary Table 1.** Mutations identified upon next generation sequencing on the mitochondrial genome of the HCT116 and HCT116+chr3 cells, using the Illumina MiSeq platform.

| **Chr** | **Position** | **Ref** | **Alt** | **Annotation** | **HCT116** | **HCT116+chr3** |
| --- | --- | --- | --- | --- | --- | --- |
| chrM | 302 | A | -C | integenic | + | + |
| chrM | 515 | G | -CA | integenic | + | + |
| chrM | 5895 | A | +C | integenic | + | + |
| chrM | 6845 | T | -C | MT-CO1 I314 frameshift | - | + |
| chrM | 16183 | A | -C | integenic | + | + |
| chrM | 16562 | C | +ATCACGATGGATCACAGGTCT | integenic | + | + |
| chrM | 16567 | C | +GATGGATCA | integenic | + | + |
