# Supplementary material for: MLH1 deficiency leads to deregulated mitochondrial metabolism
Source: Cell Death Dis. 2019 Oct 22;10(11):795. doi: 10.1038/s41419-019-2018-y (PMC6805956; doi:10.1038/s41419-019-2018-y)
Supplement: Supplementary file 2 — Supplementary Figure Legends [file 41419_2019_2018_MOESM2_ESM.docx]

**SUPPLEMENTARY FIGURE LEGENDS**

**SUPPLEMENTARY FIGURE 1**

Quantification of CI band intensity from triplicate western blots of HCT116 and HCT116+Chr3 (A) and KLE siCtrl and siMLH1 transfected cells (B) by densitometry after normalization to total protein and β-actin levels. *p<0.05, ***p<0.0005.

(C) Complex I activity was measured using an ELISA assay. Protein lysates were isolated from a panel of MLH1-deficient cell lines (HCT116, SKOV3, IGROV, AN3CA, MFE-296, SW48, A2780cp70) and the MLH1-proficient cell lines (HCT116+chr3, HT29, SW620, A2780). Equal amounts of protein were incubated to determine the activity of Complex I by measuring the oxidation of NADH to NAD+ and the simultaneous reduction of a dye leading to increased absorbance at 450 nm, over time.

(D) A panel of MLH1-deficient (HCT116, SKOV3, IGROV, AN3CA, MFE-296, SW48, A2780cp70) and MLH1-proficient (HCT116+chr3, HT29, SW620, A2780) cell lines were treated with increasing concentrations of Parthenolide (0, 0.001, 0.1, 10, 100 µM). After 4 days treatment, cell viability was measured using an ATP-based luminescence assay.
