# Supplementary figures and images for: MLH1 deficiency leads to deregulated mitochondrial metabolism
Source: Cell Death Dis. 2019 Oct 22;10(11):795. doi: 10.1038/s41419-019-2018-y (PMC6805956; doi:10.1038/s41419-019-2018-y)

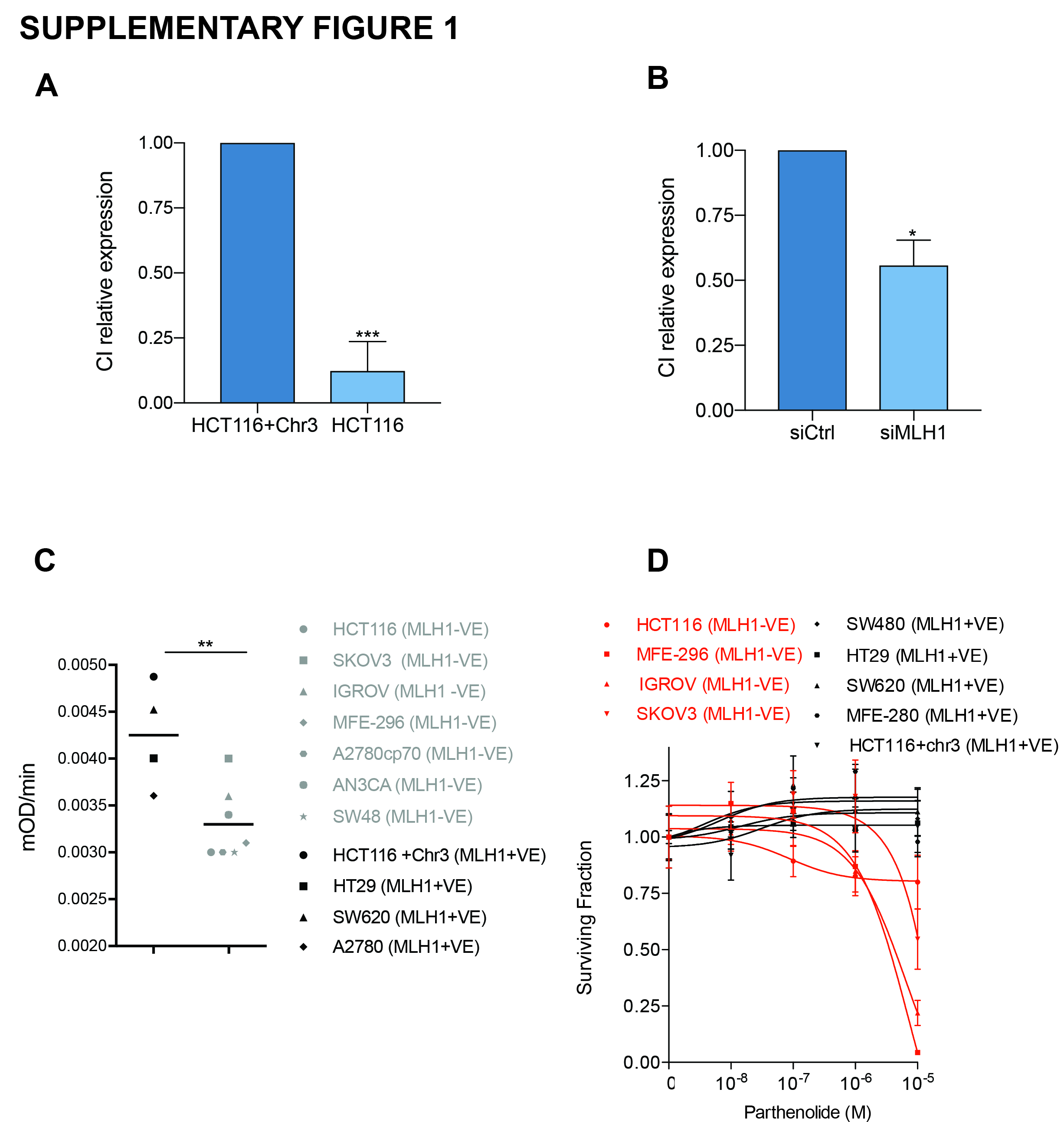

Supplement: Supplementary file 3 — Supplementary Figure 1 [file 41419_2019_2018_MOESM3_ESM.tif]
